# Recent Advances in Plant Phenolics

**DOI:** 10.3390/molecules22081249

**Published:** 2017-07-26

**Authors:** Daniel A. Jacobo-Velázquez, Luis Cisneros-Zevallos

**Affiliations:** 1 Tecnologico de Monterrey, Escuela de Ingeniería y Ciencias, Ave. Eugenio Garza Sada 2501, Monterrey NL 64849, Mexico; 2 Department of Horticultural Sciences, Texas A&M University, College Station, TX 77843-2133, USA

The scientific interest in plant phenolics as chemopreventive and therapeutic agents against chronic and degenerative diseases has been increasing since the late 1990s, when the French paradox was associated with the high intake of phenolics present in red wine [1]. Since then, research regarding the biosynthesis, biological activities, purification, and chemical characterization of phenolic compounds in different plant species has been performed. In addition, research on the stability of phenolics in food processing techniques and storage has become an area of major interest.

Twenty-one contributions (17 research and four review articles) in this special issue show some of the most recent advances in plant phenolics research. The information published includes: the chemical characterization of phenolic profiles from different plant species and the evaluation of their bioactivity; the effect of processing conditions on the phenolic composition of foods; methods for the purification of phenolics; methods for the evaluation of phenolics in blood samples; functional characterization of enzymes involved on the biosynthesis of flavonoids; and the evaluation of pre- and postharvest treatments to increase the phenolic content of different horticultural crops.

In this special issue, Navarro Hoyos et al. [2] evaluated the phenolic composition of extracts from *Uncaria tomentosa* L. (cat’s claw) from different regions of Costa Rica. Furthermore, the phenolic composition accompanied with the antiviral and antimicrobial activity of *Bombax malabaricum* [3] and *Rhoeo discolor* [4] were reported. Similarly, Nina et al. [5] compared the antimicrobial effect, total phenolic, total flavonoid, and phenolic composition of 19 samples of propolis from the Region of Maule, Chile.

Regarding the chemical characterization of phenolic compounds and the evaluation of their bioactivity, Pollio et al. [6] evaluated the polyphenolic profile and targeted bioactivity of methanolic extracts from Mediterranean ethnomedicinal plants on human cancer cell lines [6]. From the different plants evaluated, *Juniperus communis* L. and *Cotinus coggy* methanol extracts showed marked cytotoxic effect, affecting cell morphology and growth [6]. Likewise, Zhou et al. [7] elucidated the structure of two new phenolic compounds and a pair of lignin isomers from *Rhodiola crenula* and reported their antioxidant and inducing INF-γ activities.

Regarding the effect of processing on the phenolic content and bioactivity of plant foods, Yu and Beta [8] identified and measured the antioxidant properties of phenolic compounds during the production of bread from purple wheat brans, whereas Raiola et al. [9] reported the phenolic content and cytotoxicity towards cancer cells before and after the thermal processing of yellow tomatoes. On the other hand, Torres-Contreras et al. [10] reported the stability of phenolic and other bioactive compounds during storage of broccoli subjected to different cutting styles.

An emerging topic that is presented in this special issue is the development of methods to increase and/or modify the phenolic profile of horticultural crops. A review article on this topic was presented by Kaushik et al. [11]. In this context, Surjadinata et al. [12] reported the effect of different types of UV light (A, B and C) on the biosynthesis of phenolics, antioxidant capacity, and phenylalanine ammonia-lyase (PAL) activity of wounded carrots. Based on their results, the authors proposed a reactive oxygen species (ROS) mediated hypothetical mechanism explaining the synergistic effect of wounding and different UV radiation stresses on phenolics accumulation in plants. Similarly, Moreira-Rodríguez et al. [13] reported how by controlling UVA, UVB light doses and harvesting time the phenolic and glucosinolate profiles of broccoli sprouts can be manipulated. Likewise, Zhao et al. [14] used LC/MS-based metabolomics to study the effect of genotype and environment on *Salvia miltiorrhiza* roots metabolites. *Salvia miltiorrhiza* roots are broadly used as herbal medicine for the treatment of cardiovascular and cerebrovascular diseases.

Other research articles published in this special issue include the functional characterization of a dihydroflavanol 4- reductase (DFR) from fiber of upland cotton [15]. DFR is a key enzyme involved in anthocyanins and proanthocyanins biosynthesis [15]. In addition, Bongartz et al. [16] reported the mechanism of green discoloration of sunflower extraction meal, which was associated with the production of benzacridine by the reaction between *o*-quinones (produced from chlorogenic acid) with nucleophiles present in proteins.

In relation with the purification of phenolic compounds and their further applications as food additives, Aguilar and Hernández-Brenes [17] explored the feasibility of modifying phenolic profiles of thyme extracts, by use of chromatographic resins, to obtain phenolic extracts capable of enhancing anthocyanin color and stability in the presence of polyphenol oxidase activity. On the other hand, Guajardo-Flores et al. [18] reported an adequate maltodextrin-extract ratio for the spray-drying process to produce nutraceutical capsules containing flavonoids and saponins from black bean extracts with proven health benefits. Likewise, Martínez-Huélamo et al. [19] reported a sensitive and rapid UHPLC-MS/MS for the analysis of tomato phenolics in human biological samples.

Other review articles published in this special issue include a contribution about insoluble-bound phenolics, explaining their localization and biosynthesis in plant cells, as well as their metabolism in human digestive system and corresponding bioactivity [20]. Likewise, Serna and Martínez [21] reviewed the phenolic and polyphenolic metabolites described in literature for several genera of the Metastomataceae family, which is the seventh largest of flowering plants.

Finally, Santana-Gálvez et al. [22] reviewed the recent advances on the dual application of chlorogenic acid as a food additive and a nutraceutical against metabolic syndrome. The authors proposed a model for preventive and therapeutic effects of chlorogenic acid or chlorogenic acid-rich foods and supplements over metabolic syndrome based on in vivo studies and clinical studies.
